# What Is the Ideal Blood Pressure Treatment Target for Primary Prevention and Management of Atrial Fibrillation?

**DOI:** 10.3389/fcvm.2020.586183

**Published:** 2020-11-27

**Authors:** Xianghong Meng, Xiaoyong Xu

**Affiliations:** ^1^Ningbo College of Health Sciences, Ningbo, China; ^2^Department of Cardiovascular Disease, Ningbo Medical Center Lihuili Hospital, Ningbo, China

**Keywords:** hypertension, atrial fibrillation, risk factor, guideline, cardiovascular outcome

## Introduction

Atrial fibrillation (AF), which is the most common cardiac arrhythmia, accounts for approximately one-third of hospitalizations for cardiac rhythm disturbances. Each year, more than 5 million people develop AF on this planet, with a significant impact on health care (1). AF is independently associated with significant morbidity and mortality, including a four- to five-fold increased risk for stroke, a three-fold risk for heart failure, a two-fold increased risk for dementia, and a significant risk for premature death (1, 2).

For all patients with AF, the current guidelines report that we think about not only stroke prevention, rate/rhythm control, but also risk factor modification (3, 4). Evidence accumulated in recent years indicates that risk factor management is associated with significant clinical and cost-effectiveness benefits on AF. Many potential modifiable risk factors that contributed to the underlying atrial substrate and, therefore, to AF development have been described, such as hypertension, obstructive sleep apnea, obesity, and diabetes (1, 3, 4). Due to its high prevalence in the general population, hypertension is the most common modifiable risk factor associated with AF (5), and poorly controlled blood pressure (BP) has been associated with elevated AF risk (6, 7). A study showed that hypertension (defined as systolic blood pressure (SBP) >140 mmHg or diastolic blood pressure >90 mmHg or receiving antihypertensive treatment) increased the risk of AF by 56% (8). In a recent large, observational study in close to 1 million Koreans, Kim et al. showed that the incidence of incident AF gradually increased in response to increased BP (9). Even “pre-hypertension” (SBP 130–139 mmHg) was associated with incident AF (9). Besides, hypertension increases the risk of cardiovascular events in patients with AF. In the ROCKET AF trial, a prospective, multicenter, double-blind, randomized controlled trial in patients with non-valvular AF and moderate to high risk of stroke, 90.5% of 14,256 anticoagulated patients had hypertension (10). Vemulapalli et al. found that the risk of stroke or systemic embolism in these patients increased significantly for every 10-mmHg increase in screening SBP (10). In the ARISTOTLE trial of more than 18,000 patients (mean age 70 years) with non-valvular AF, patients with uncontrolled BP at any point during the 2-year trial were associated with a 53% increased risk of ischemic stroke, a 38% increased risk of myocardial infarction, and an 85% increased risk of hemorrhagic stroke (11). These data underscore the importance of intensive antihypertensive therapy to achieve intensive BP control to reduce the risk of new-onset AF and improve the outcome of AF. However, the ideal BP treatment goals for improving the substrate and outcome of AF remain to be defined.

## Mechanisms of Hypertension-Related AF

When someone has high BP, the heart needs to pump against that stress hour after hour, day after day. The stress results in left ventricular (LV) hypertrophy, decreased diastolic function with impaired LV filling, with subsequent rising left atrial pressures with left atrial hypertrophy and enlargement, increased atrial fibrosis, and slowing of intra-atrial and interatrial elec-velocities (12, 13). Such a distortion of atrial anatomy and physiology ultimately predisposes to AF. In a study of patients with essential hypertension free of other cardiovascular conditions (*n* = 2,482), the incidence of AF was 0.46% per year, and age or increased LV mass was the only independent predictors of incident AF during a 5-year follow-up (14). Left atrial pressure also increases with ischemic or valvular heart disease and myopathies that are often associated with systemic hypertension, potentially leading to AF (15). Atrial remodeling induced by hypertension is known as atrial cardiomyopathy, which is described in detail in a recent expert consensus (16).

The abnormal atrial substrate is reversible. A study demonstrated that the renin–angiotensin–aldosterone system blockers could improve atrial electrical and structural remodeling (17). BP reduction following renal sympathetic denervation (RSD) in patients with resistant hypertension was associated with the global atrial conduction improvement (18).

## Long-Term Benefit of Goal-Directed BP Reduction in AF

BP control can reduce the occurrence and progression of AF. In patients referring to AF ablation, a previous study demonstrated that poorly treated hypertension almost wholly eliminated the effect of AF ablation (19). Conversely, the ARREST-AF trial showed that aggressive risk factor management, including achieving a target BP of <130/80 mmHg at least 80% of the time, could improve the long-term success of AF ablation (20). Similarly, in a recent randomized trial of 302 patients with hypertension and symptomatic AF randomized to AF ablation with or without RSD, RSD was associated with a significant reduction in BP and AF recurrence at 12 months (21). Besides, in a *post hoc* analysis of the LIFE study of more than 8,831 patients (mean age 67 years) with LV hypertrophy, compared with placebo (SBP ≥142 mmHg) during a mean of 4.6 years follow-up, antihypertensive treatment targeting SBP <140 mmHg and SBP <130 mmHg was associated with a 24 and 40% lower risk of new-onset AF, respectively (22). The benefit for the achievement of an SBP of ≤ 130 mmHg in the prevention of AF was also evidenced in the prespecified secondary outcome of the Cardio-Sis trial, which demonstrated that new-onset AF occurred in 3.8% of patients in the usual control group (SBP <140 mmHg) and 1.8% of patients in the tight control group (SBP <130 mmHg) (23).

BP control can also improve outcomes in AF patients. Patients with both AF and hypertension had an increased risk of stroke when compared with patients with either condition alone (24). Active treatment lowering mean BP by 7.3/3.4 mmHg was associated with a 34% reduction in stroke (25). In a retrospective study from China, conducted in anticoagulated hypertensive patients with AF, those who achieved a target BP <130/80 mmHg showed a lower incidence of ischemic stroke and a similar risk of major bleeding and intracranial bleeding than patients with higher BP values (26).

## What Is the Ideal BP in the Prevention of AF?

The role of hypertension as a modifiable risk factor for AF is established but still incompletely known. In particular, what is the ideal BP treatment target from the perspective of improving the substrate of AF? For this objective, Parcha et al. conducted a *post hoc* analysis aiming to study the influence of a more intensive reduction in SBP to <120 mmHg on incident AF. A total of 8,549 participants in the SPRINT Trial were eligible for primary analysis (27). The results showed that there was a similar rate of new-onset AF in intensive (target SBP <120 mmHg) and standard (target SBP <140 mmHg) treatment arms (27). That is, a more intensive reduction in SBP to <120 mmHg would not reduce new-onset AF compared with the goal SBP <140 mmHg. This result was consistent with the results of Thomas et al., who conducted a case-controlled study of patients undergoing treatment for hypertension (28). Compared with a reference SBP level of 120–129 mmHg, both SBP ≥150 mmHg and SBP <120 mmHg were at higher risk of incident AF in multivariable logistic regression models (28). The J-curve relationship between BP and new-onset AF was also observed in the control of hypertension in patients undergoing catheter ablation. In a randomized study, Parkash et al. randomly assigned 184 patients with AF and a BP >130/80 mmHg to aggressive BP (target <120/80 mmHg) or standard BP treatment (target <140/90 mmHg) prior to their scheduled AF catheter ablation (29). This study found that targeting SBP to <120 mmHg did not reduce atrial arrhythmia recurrence after catheter ablation of AF during a median follow-up period of 14 months (29).

These findings suggest that a target SBP of 120–129 mmHg may be the ideal SBP in the prevention of AF. However, this aspect was not confirmed in a *post hoc* analysis of the Women's Health Study, which showed a negative linear association between BP and the risk of incident AF during a median follow-up period of 14 years (6). Their time-updated analyses suggested that the substantial risk of incident AF reductions might be obtained even if SBP was ≤120 mmHg (6). Optimal BP targets to improve the substrate of AF need to be verified in future randomized trials.

## What Is the Ideal BP for Improving the Outcome of AF?

Whether stricter BP control provides additional protection in patients with both AF and hypertension is unclear. Kim et al. analyzed data in 298,374 Korean adults with hypertension and non-valvular AF to determine the optimal BP threshold in patients with AF (30). They reported that in comparison with a reference BP level of 120–129/80 mmHg, patients with BP >130/80 mmHg or <120/80 mmHg presented significantly higher risks of major cardiovascular events, including intracranial hemorrhage, ischemic stroke, myocardial infarction, and heart failure requiring hospitalization (30). In the SPRINT trial, ~35% (*n* = 281) of participants with preexisting AF achieved the BP level of <120/80 mmHg at 3 months (27). Similarly, SBP <120 mmHg was not associated with a lower risk of adverse cardiovascular events compared with those with preexisting AF and BP of ≥120/80 mmHg (27). That implies that a BP of 120–129/80 mmHg might be the optimum BP for patients with AF undergoing hypertension treatment.

## BP Treatment Targets in Patients With AF Across Major Hypertension Guidelines

Table 1 compares eight sets of hypertension guidelines last updated between 2016 and 2019 (15, 31–38). With gaps in the evidence base, expert panels disagree on whether guidelines of hypertension should list patients with AF as a specific population and provide a recommendation for BP goal to be achieved in this specific circumstance. The 2019 Japanese Society of Hypertension guidelines recommend that an SBP target of 130 mmHg is effective in preventing the new onset of AF, cardiovascular events (e.g., stroke), and anticoagulation-related bleeding in patients with both AF and hypertension (32). The 2018 European Society of Cardiology and European Society of Hypertension guidelines and 2018 Chinese Guidelines of Hypertension suggest that BP treatment targets in individuals with AF should be at least <140 mmHg, and that <130 should be considered if tolerable (34, 38). The 2018 Korean Society of Hypertension guidelines state that in patients with AF and hypertension, lowering BP can decrease the incidence of fatal bleeding during antithrombotic treatment. However, BP should be maintained above 120/70 mmHg (37). The 2017 American Heart Association/American College of Cardiology (AHA/ACC) guidelines of hypertension recommend BP targets <130/80 mmHg in all hypertensive patients with different recommendation levels according to the risk of cardiovascular disease (15). The Canadian, United Kingdom, and Australia guidelines of hypertension neither list patients with AF as a specific population nor suggest a BP target goal in this situation (31, 33, 35).

**Table 1:** Blood pressure treatment targets in patients with atrial fibrillation across major hypertension guidelines.

**Guideline**	**Population**	**BP treatment targets**
2019 UK NICE	Do not list patients with AF as a specific population	–
2019 JSH	Patients with AF	<130 mmHg
2018 Canada	Do not list patients with AF as a specific population	–
2018 ESC/ESH	Patients with AF	<140 mmHg (well-tolerated <130 mmHg)
2018 South Korea	Patients with AF	Not <120/70 mmHg
2018 China	Patients with AF	<140 mmHg (well-tolerated <130 mmHg)
2017 ACC/AHA	General (including patients with AF)	<130/80 mmHg
2016 Australia	Do not list patients with AF as a specific population	–

*UK, United Kingdom; NICE, National Institute for Health and Care Excellence; JSH, Japanese Society of Hypertension; ESC, European Society of Cardiology; ESH, European Society of Hypertension; AF, atrial fibrillation; BP, blood pressure; ACC, American College of Cardiology; AHA, American Heart Association*.

## The Direction of Future Research

Note, though, that new-onset AF was detected by hospital admissions, medical records, and the scheduled study ECGs in the trials mentioned in this paper. The results of intermittent ECG may not always reflect the “true” incidence of new-onset AF. AF can come and go, particularly early in the course of the disease. It is not uncommon for paroxysmal AF to go undetected in intermittent monitoring, which results in a low detection rate of new incident AF cases. Even an ECG taken over a longer period (>24 h) using a Holter monitor does not always lead to a reliable diagnosis of existing AF. In a study of 82 outpatients ≥65 years old with hypertension, diabetes mellitus, and no history of AF or any other cardiovascular, Philippsen et al. found that only 11.8% of patients with asymptomatic AF, which was detected by implantable loop record (ILR), had AF episodes on a 72-h Holter monitoring (39). Another difficult issue is regarding BP measurement in AF. In the presence of AF, variations in ventricular filling time, stroke volume, and contractility may lead to increased beat-to-beat BP variability, which may affect BP estimation using both the auscultatory and oscillometric methods (40). All these would critically impact the result of correlation analyses. An optimal approach to assess the impact of hypertension on AF in large population-based cohort studies remains unclear. Given insufficient sensitivity in detecting sporadic arrhythmias (such as paroxysmal AF) by intermittent monitoring, a continuous rhythm monitoring strategy (such as ILR), although invasive, overcomes many of the limitations of intermittent monitoring in assessing the rate of incident AF and helps evaluate the real effect of hypertension on AF. New mHealth technologies may also provide a promising infrastructure for a more objective and longitudinal assessment of new-onset AF. For this reason, the AF detection function of wearable devices, which receive extensive attention from device manufacturers and cardiologists, has become a focus of research (41). Although current guidelines do not recommend automated BP monitor in the presence of AF (31, 34, 35, 42), the research results about its ability of AF detection during routine BP measurement are promising and worth further studies (43, 44).

## Conclusion

Hypertension is a powerful trigger for AF and increases the risk of cardiovascular events in patients with AF. To date, the available evidence supports targeting BP at guideline-recommended levels for primary prevention and management of AF, with J- or U-curve relationship existing between BP and substrate/outcome of AF.

## Author Contributions

XM and XX wrote the manuscript, conceptualized the idea, and reviewed and approved the final manuscript for publication. Both authors contributed to the article and approved the submitted version.

## Conflict of Interest

The authors declare that the research was conducted in the absence of any commercial or financial relationships that could be construed as a potential conflict of interest.
